# EMPOWER‐PD: Evaluation of an 8‐Week Co‐Designed Self‐Management Program in People With Parkinson’s Disease and Their Caregivers: An Intervention Study With Long‐Term Follow‐Up

**DOI:** 10.1155/padi/9413261

**Published:** 2025-12-06

**Authors:** Trine Hørmann Thomsen, Sara Lungby Skovbølling, Maria Brønden, Jakob Frederiksen, Maja Hedegaard Lauritzen, Marcus Dalsgaard, Bo Biering-Sørensen

**Affiliations:** ^1^ Movement Disorder and Pain Research Center, Department of Neurology, Copenhagen University Hospital, Copenhagen, Denmark, gentoftehospital.dk; ^2^ Department of Brain and Spinal Cord Injuries, Copenhagen University Hospital, Copenhagen, Denmark, gentoftehospital.dk; ^3^ Department of Neurology, Bispebjerg-Frederiksberg University Hospital, Copenhagen, Denmark; ^4^ Sano specialized Rehabilitation Center, Skælskør, Denmark; ^5^ Department of Public Health, Faculty of Health and Medical Sciences, University of Copenhagen, Copenhagen, Denmark, ku.dk

**Keywords:** coping strategies and goal-setting, empowerment, Parkinson’s disease, symptom burden

## Abstract

Empowerment and implementation of self‐management strategies are vital components in the future care of chronic, neurological patient groups, including people with Parkinson’s disease (PD). This study aimed to evaluate the feasibility and effectiveness of an 8‐week self‐management intervention designed for people with PD (PwP) with follow‐up assessments at 3 and 6 months postintervention. The program focused on developing self‐management skills, coping strategies, disease education, mindfulness, exercise routines, and individual goal‐setting tools. Objective measurements using Parkinson’s KinetiGraph™ (PKG) were employed to monitor motor symptoms, immobility levels, and burden of nonmotor symptoms (self‐reported in the PKG portal). Participants, serving as their own controls, were recruited from movement disorder clinics, private neurologists, and a specialized rehabilitation center. Primary endpoints were change in Health Education Impact Questionnaire (HeiQ) and Health Literacy Questionnaire (HLQ‐14) scores, assessed at baseline and at 3‐ and 6‐month follow‐up. Secondary endpoints included changes in quality of life, self‐efficacy, motor symptoms (PKG data), nonmotor symptoms, contacts to the clinics 6 months before and after the intervention, and time spent with immobility. Eighty PwP completed the program, and 59 caregivers attended the educational sessions. Statistically significant changes were found between baseline and follow‐up in the motor symptom burden, self‐efficacy, and reduced clinical contacts. Although no statistical changes were observed in health literacy, quality of life, and nonmotor symptoms, sustained positive trends were observed. The findings suggest that the program may enhance empowerment and self‐management strategies in PwP, particularly in self‐efficacy level, managing motor symptoms, and less need of contact to the clinics.

## 1. Introduction

Parkinson’s disease (PD) is a progressive neurodegenerative disorder characterized by a wide range of motor and nonmotor symptoms (NMS) that can significantly impact the quality of life (QoL). As the disease progresses, people with PD (PwP) often experience increasing challenges in managing their symptoms and maintaining independence [1, 2]. Additionally, adherence may decline as cognitive impairment progresses, reducing patients’ ability to manage, initiate, and administer doses and further impairing memory [3]. Self‐management programs have emerged as a promising approach to empower individuals with chronic conditions, including PD, to take an active role in managing their health and engage in decision‐making with healthcare professionals (HCP) [4, 5].

Managing both motor and NMS plays a significant role in PwP′ QoL, as daily life can be marked by considerable unpredictability depending on the severity of symptoms from day to day [4, 6]. Although PwP require regular consultations, including symptom evaluation and adjustment of medical treatment, the daily care and management of symptoms are carried out by the individuals and their relatives themselves, and they inevitably take on the primary responsibility for daily self‐management. Therefore, both parties must develop emotional, cognitive, and practical skills to adapt to life with PD [7, 8].

Self‐management is defined as “the performance of activities that individuals initiate and carry out on their own behalf to maintain life, health, and well‐being” [9]. The concept refers to both cognitive processes, including the observation and assessment of symptoms and daily activities, and to a person’s ability to manage symptoms, treatments, lifestyle changes, psychosocial stress, and other consequences of the disease, in collaboration with family, community, and HCPs [9, 10]. The ability to adjust and engage in self‐care activities is crucial, and managing the disease is considered an essential competence in chronic illnesses [4, 10].

In a scoping review from 2021, barriers to access healthcare services for PwP at two levels, person level and system level, were identified [11]. Barriers on a person level included skills to seek healthcare services, which relate to personal autonomy and capacity to seek care, the ability to engage in care services, be involved in own treatment and decision making, poor health literacy, and cost, and finally availability of relevant services at a system level [11]. Specifically poor health literacy negatively affected both access to care services and also health‐related outcomes. Communication skills and self‐efficacy related to lack of skills to communicate, e.g., problems and needs, were also identified [11]. Consistent with this rationale, a recent systematic review and meta‐analysis in PD reports benefits of self‐management interventions on outcomes such as QoL and self‐efficacy [12].

Wannheden et al. identified six digital self‐management functionalities based on input from PwP and HCPs in a co‐design process inspired by participatory design principles [13–15]. These were self‐tracking, previsit forms, graphical visualization, clinical decision support, self‐care recommendations, and asynchronous communication, to support collaboration between patients and HCPs [13]. We used these functionalities as a design scaffold for our intervention and, in a co‐design process, adapted them into a blended format that combined digital elements; symptom and physical‐activity tracking with graphical feedback—a need especially expressed by the PwP—and structured daily metrics visualized to facilitate shared decisions, with behavioral components prioritized by the stakeholders, such as coping strategies, disease education, mindfulness practices, physical activity, and individualized goal‐setting during previsits. This approach acknowledges the diversity of intervention formats and outcomes in PD self‐management [12, 16], which is an aspect that needs to be highlighted.

**Figure 1:**
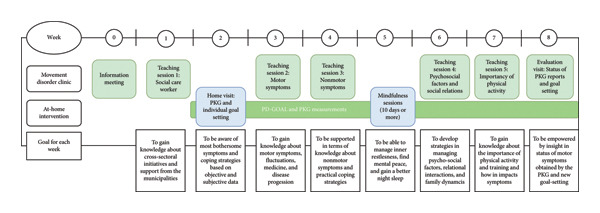
Overview of the self‐management program, including sessions and goals for each week.

In this study, we tested the 8‐week blended self‐management program that implements and adapts Wannheden’s six digital functionalities within a co‐designed format including perspectives of PwP, caregivers, and HCPs. The program was designed to address key aspects of disease management, including the development of coping strategies, disease education, mindfulness practices, and physical activity, as well as individual goal‐setting [17]. The overall program is displayed in Figure 1. The aim of the study was to evaluate the feasibility and effectiveness of the program in an intervention with long‐term follow‐up for PwP and caregivers.

## 2. Methods and Materials

### 2.1. Study Design

This study is a multicentre, single‐arm, follow‐up study to evaluate the long‐term effect of the self‐management program.

### 2.2. Objective and Hypothesis

The objective was to evaluate the effect of a co‐designed 8‐week self‐management program including follow‐up assessments at 3 and 6 months post‐intervention using objective measurements and self‐reported questionnaires.

We hypothesized that participation in an 8‐week co‐designed self‐management program, followed by 3‐ and 6‐month post‐intervention with‐in subject assessments, would improve in health education impact, health literacy, QoL, and self‐efficacy among PwP and, additionally, that the program would reduce the frequency of clinic visits and demonstrate measurable improvements in motor symptom and NMS management.

### 2.3. Participants

Participants were recruited from two movement disorder clinics (MDCs) at Copenhagen University Hospital, Rigshospitalet, Bispebjerg‐Frederiksberg University Hospital, a specialized rehabilitation center, and private neurologist and physiotherapist clinics between October 2022 and January 2024.

Eligibility criteria included diagnosis of Idiopathic PD, Hoehn and Yahr stage of disease (1–3), the ability to provide informed consent, and the physical and cognitive capacity to participate in the self‐management program, evaluated by clinicians at the MDCs. Exclusion criteria were significant cognitive impairments (based on a Montreal Cognitive Assessment score < 24) [18] and comorbidities that would interfere with participation.

Caregivers were invited but not required to attend sessions, and PwP patients could enroll independently. The rationale was to reflect real‐world PD self‐management, where care partners often support treatment routines, symptom monitoring, and communication with HCPs and may enhance transfer of skills to daily life.

### 2.4. Sample Size

The sample size was determined based on the primary outcome, the Health Education Impact Questionnaire (HeiQ) [19] using a power calculation to detect a minimal clinically important difference (MCID). There is no universally established MCID for either HeiQ specifically in PD populations. However, Osborne et al. [20] reported that 0.4 to 0.6 SD change in a specific HeiQ domain of the baseline score is a practical estimate of meaningful change, especially in short interventions (8–12 weeks). The power analysis accounted for expected variability in HeiQ scores and a 10% dropout rate, ensuring sufficient statistical power to evaluate the intervention’s effect on participants being their own controls. A sample of minimum 82 PwP was calculated.

### 2.5. The Self‐Management Program

The initial co‐creation process in developing the self‐management program, leveraging the diverse expertise and perspectives of the target group, led to an intervention that was comprehensive and holistic in addressing the multifaceted challenges faced by PwP [17]. Each session was structured around specific goals formulated based on these needs.

The 8‐week self‐management program included weekly educational sessions designed to enhance understanding of PD and empower participants in integrating self‐management strategies into everyday life. The weekly educational sessions consisted of access to municipality services, focus on motor symptoms and NMS, medication management, the importance of physical activity, the effects of mindfulness, psychological aspects, and individual goal‐setting. In each session, workshops between the participants in small groups, were integrated, focusing on specific themes determined by the participants in the co‐design phase [17].

#### 2.5.1. Individual Goal Setting

In the second week of the program, the project manager conducted home visits with each participant. During these visits, the “PD GOAL” tool (Figure S1), developed during the co‐design phase with input from PwP and caregivers [17], was used to collaboratively establish personalized goals. These goals focused on addressing the individual participant’s most severe and disruptive symptoms in daily life, including motor symptoms, NMS, and psychosocial challenges. Regular check‐ins throughout the program provided ongoing motivation and support and helped the participants stay on track to achieve their goals while adapting strategies as needed.

#### 2.5.2. Integration of Wearable Data

The program incorporated measurements using the PKG watch, as PwP involved in the co‐design process expressed a desire for a tool to objectively track their motor symptoms and provide measurable data rather than relying solely on subjective perceptions of improvement or worsening. The PKG provides objective, continuous, and automated remote (home‐based) assessment of motor and night‐time symptoms of PD, based on average recording data from the period. During the home visit in week two, the watch was put on the dominant affected wrist of the participant. The participants were also provided with a small docking station enabling them to download their data and charge the watch at the same time. The PKG report provided mean scores with cut‐off parameters for bradykinesia (BK), dyskinesia (DK), tremor, activity at night, and medication adherence [21–23]. Various patterns of off periods in terms of timing, duration, and severity could be visually recognized by the participants, accessing a patient portal to log daily symptoms, including registration of NMS burden, well‐being, and activity levels, identifying their best and worst times of day, and fostering a more comprehensive understanding of their condition. Participants wore the PKG watch during the 8‐week intervention and for 14 days at each follow‐up assessment (3 and 6 months post‐intervention).

#### 2.5.3. Self‐Management Skills

The sessions equipped participants with practical techniques to take an active role in managing their health and disease symptoms. Topics included medication adherence, knowledge about motor symptoms and NMS, symptom tracking, and effective communication with healthcare providers. Also, a focus on psychosocial factors, such as loss of identity, role dynamic and communication in the family, and interacting in social relations, was integrated in the sessions. Summarized, the participants were taught and engaged in techniques to help them take control of their health and manage their symptoms more effectively. Often, the dialog in the small workshops included approaches and strategies to manage stress, anxiety, and depression. Techniques such as behavioral strategies and peer support were also highlighted.

#### 2.5.4. Mindfulness Session

This session introduced mindfulness techniques, such as meditation and breathing exercises, aimed at reducing stress and promoting mental clarity. Participants were introduced to mental coping strategies for dealing with the unpredictable nature of PD symptoms and their impact on daily life. The mindfulness session was developed by a physiotherapist specialized in mindful breathing, and a customized virtual link was created for the participants in the program, who were encouraged to integrate the session minimum one time per day during the program or at least for 10 days in week 5 and 6.

#### 2.5.5. Physical Activity and Exercise

The participants were provided with guidance on maintaining and increasing physical activity levels. During the session, the importance of staying physically active to maintain mobility and overall health was emphasized and also it was emphasized that “every move counts” highlighting the vision from the World Health Organization (WHO) [24]. Tailored exercise routines were suggested depending on the raised questions from the groups. Mostly it included strategies on improving basic mobility, balance, strength, and flexibility.

### 2.6. Outcome Measures

#### 2.6.1. Primary Endpoints

Changes in the HeiQ [19] and HLQ‐14 [20] were primary endpoints. The changes in scores from baseline to 3‐ and 6‐month follow‐up were assessed.

HeiQ [19] is a validated tool, assessing the impact of health education and self‐management interventions on individuals’ health‐related outcomes. The scale measures different aspects of health education impact, such as positive and active engagement in life, emotional well‐being, self‐monitoring, constructive attitudes and approaches, social integration and support, and health services navigation. The total score for each subdomain of the HeiQ was calculated by summing the responses to the individual items on a 4‐point Likert scale (1–4) within that subscale, ranging from 40 to 160 points summarized. A low score indicates a lesser impact or perception of the effectiveness of health education interventions.

Changes in health literacy were assessed using the HLQ‐14 instrument [20], a validated tool designed to evaluate participants’ ability to access, understand, appraise, and apply health‐related information, as well as communication with HCPs, navigation of healthcare systems, and the capacity to make informed decisions about personal health [25]. The scale is divided into two sections, functional health literacy (FHL), focusing on the ability to obtain and process basic health information, and, the second part, communicative and critical health literacy (CCHL), which evaluates the ability to interpret, critically analyze, and apply health information in decision‐making. Both parts are assessed using a five‐point Likert scale (0–5). Part one score (23 subitems) ranges from 0 to 92. Part two score (21 subitems) ranges from 0 to 84. Low scores indicate less health literacy.

#### 2.6.2. Secondary Endpoints

Secondly, changes in QoL, as measured by the Parkinson’s Disease Questionnaire (PDQ‐39) [26] and self‐efficacy using the general self‐efficacy scale (GSES) [27], were assessed. NMS burden was assessed using either the patient portal or the analog questionnaire, Nonmotor Symptom Questionnaire (NMSQuest) [28]. The analog scale was used if the participants preferred that version. Additionally, the difference in numbers of contacts/phone calls and visits/consultations at the MD clinics were registered from the electronic patient journal 6 months pre‐intervention and 6 months post‐intervention.

#### 2.6.3. Motor Symptom Scores and Immobility Levels (Algorithm Data from the PKG)

Change in BKS, change in DKS, and time spent with immobility, as recorded by the PKG, were assessed. The score is categorized as mild to moderate or severe BK. If the BKS was between 25 and 39.3, it was considered mild to moderate BK. If the BKS was between 39.3 and 80, it is considered severe BK. Cut‐off values have been determined by comparing patient data to a control group [21].

### 2.7. Data Collection

Data collection was conducted at baseline and 3‐month and 6‐month follow‐up. Motor symptom scores and immobility levels were tracked at baseline (after first week), post‐intervention, and during the 3‐ and 6‐month follow‐up periods.

Descriptive data included age, gender, years with PD, Hoehn and Yahr (H&Y) stage, levodopa equivalent dose, cohabitation status, education level, comorbidities, and independence in activities of daily living (ADL).

Additionally, at the end of the 8‐week program, participants received an evaluation survey to assess their experience and outcomes, with the possibility to include open, qualitative descriptions.

Caregiver‐reported outcomes were not collected; all primary and secondary outcomes pertained to patients.

Validated Danish versions of all instruments were used according to their manuals. Internal consistency was evaluated in the present sample for each multi‐item scale/subscale (ordinal Cronbach’s *α*). Missing items were handled per instrument guidance (proration when ≤ 10% items were missing on a scale, otherwise treated as missing for that scale).

### 2.8. Statistical Analysis

The statistical analyses were performed with the use of R software version 2023.06.1 + 524 (R project for statistical computing).

Descriptive variables were analyzed using summary statistics to provide an overview of the study population and baseline characteristics. Continuous variables were reported as means with standard deviations, while categorical variables were presented as frequencies and percentages.

A repeated measures ANOVA was conducted to assess changes over time for each outcome variable. This method was chosen to account for within‐subject correlations and to test if there were statistically significant differences in participant scores across the measurement points. Boxplots were used for visualization, providing a clear depiction of score distributions across time points for each outcome measure. Statistical significance was determined using a *p* value threshold of 0.05, with results considered significant if *p*  <  0.05.

### 2.9. Ethics

The study was granted ethical approval from the Capitol Region Research Ethics Committee, Copenhagen (IRB: H‐22013230), and was registered on the internal data portal, *Privacy* (P‐2021‐796).

## 3. Results

The recruitment process for the study, conducted between October 2022 and January 2024, involved collaboration with multiple organizations across two Danish regions. A total of 96 candidates were screened based on predefined inclusion and exclusion criteria. Candidates were recruited from six sites: Rigshospitalet (*n* = 51), Bispebjerg‐Frederiksberg Hospital (*n* = 22), Sano Rehabilitation Center (*n* = 3), private practice physiotherapy (*n* = 6), self‐initiated contact (*n* = 11), and private practice neurologist (*n* = 3).

**Figure 2:**
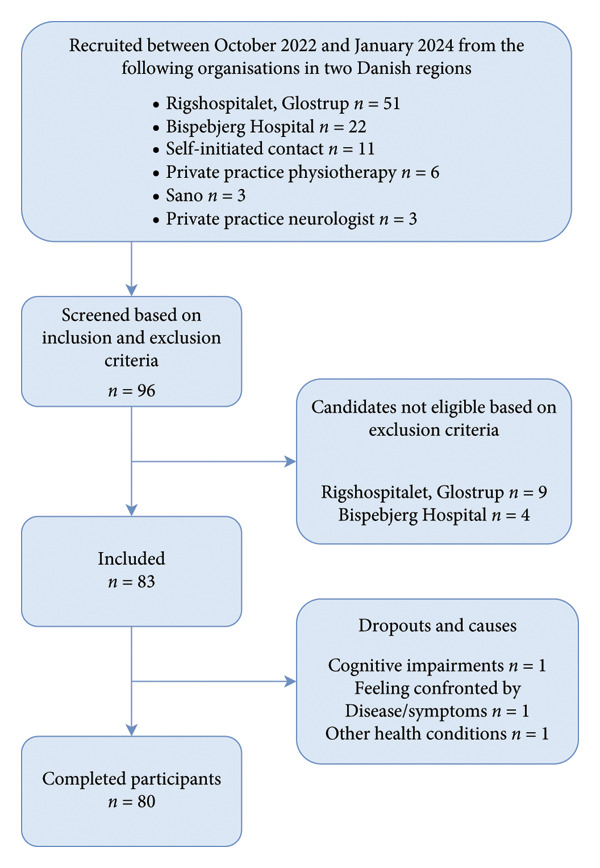
Flowchart of the recruitment and inclusion process.

Following screening, 83 participants met the inclusion criteria and were included in the study, while 13 candidates were excluded due to not meeting eligibility requirements (Rigshospitalet: *n* = 9; Bispebjerg‐Frederiksberg Hospital: *n* = 4). Subsequently, three participants dropped out due to cognitive impairment (*n* = 1), confrontation (*n* = 1), or other health conditions (*n* = 1). This resulted in a final cohort of 80 participants who successfully completed the study (Figure 2). Follow‐up measurements were finalized in September 2024. In total, 59 caregivers (78% spouses, 16% children, and 6% in‐kind relatives) followed the program.

Table 1 provides an overview of the demographic, clinical, and functional characteristics of the participants. The cohort consisted of 80 individuals, with 53.8% being men and 46.2% women. The mean age of participants was 64.3 years, ranging from 43 to 86 years. The average disease duration was 6.0 years, spanning 1–23 years. Based on the H&Y scale, 36.3% were in stage 1, 42.5% in stage 2, and 21.2% in stage 3, reflecting varying levels of disease severity. Cognitive function, assessed by the MoCA score, showed a mean score of 27.8, with a range of 24–30, indicating relatively preserved cognitive abilities in the group.

**Table 1:** Characterization of the 80 participants based on sociodemographics and clinical tests.

Gender	*N*	%
Man	43	53.8
Women	37	46.2
Age, mean/SD, range	64.3/9.8	43–86
Disease duration (years), mean/SD, range	6.0/4.2	1–23
Hoehn and Yahr score	*N*	%
1	29	36.3
2	34	42.5
3	17	21.2
MoCA score, mean/SD, range	27.8/1.5	24–30
Living alone	*N*	%
Yes	12	15.0
No	68	85.0
Working status	*N*	%
Yes	16	20.0
Yes, flex job	11	13.8
Early retirement	25	31.2
Retired	28	35.0
Education	*N*	%
7 or less years in school	4	5.0
8–11 years in school	18	22.5
Graduate degree	23	28.8
Higher education	35	43.7
Home nursing	*N*	%
Yes	2	6.3
No	75	93.7
Independence in ADL	*N*	%
Yes	66	82.5
No	14	17.5
Comorbidity	*N*	%
Yes (1–2 chronic conditions)	25	31.3
No	42	52.5
More than 2 chronic conditions	13	16.2

Most participants (85.0%) lived with a spouse, and 82.5% reported independence in ADL. Regarding employment status, 35.0% were retired, 31.2% were on early retirement, and 20.0% were actively working, including 13.8% in flex jobs. Education levels revealed a diverse background, with 43.7% having higher education and 28.8% holding graduate degrees. Comorbidity analysis showed that 52.5% had no chronic conditions beyond PD while 31.3% had one to two additional conditions and 16.2% had more than two. A small proportion (6.3%) required home nursing.

This characterization highlights a group of relatively well‐functioning participants with preserved cognitive function and varying levels of education and comorbidities.

### 3.1. Results of the Primary Outcomes

**Figure 3:**
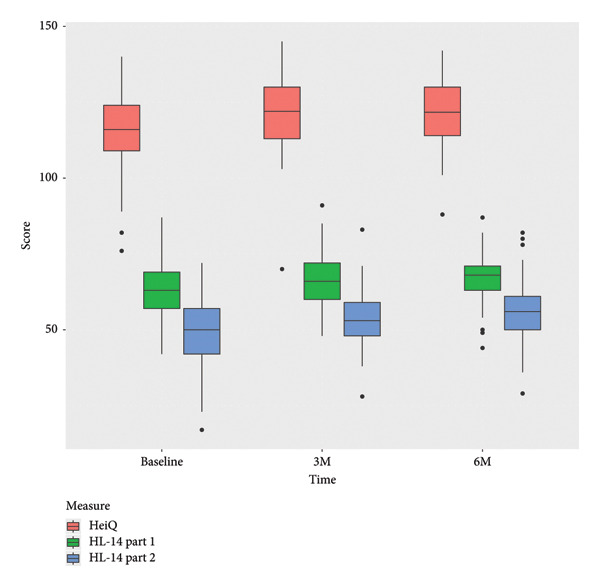
Boxplots of the primary outcome measures, HeiQ and HLQ‐14 at baseline and 3‐ and 6‐month follow‐ups.

The mean score in HeiQ improved from baseline to 3 months follow‐up but leveled out between 3‐ and 6‐month follow‐ups (Figure 3). These findings indicated sustained improvements in HeiQ scores at baseline (mean 92.8) and data collection at 3 months (mean 111.6) and 6 months (mean 105.8); however, the difference from baseline to 6‐month follow‐up in HeiQ score showed no statistically significant difference (*p* = 0.067).

The results showed an improvement in mean HL scores. However, for HLQ‐14 part 1, which assesses participants’ ability to access and understand health information, the within‐subject difference was not statistically significant (*p* value = 0.101) and, similarly, HLQ‐14 part 2, which evaluates the ability to appraise and apply health information, showed an improvement in mean score but no statistical significance (*p* = 0.337). See Figure 3.

### 3.2. Results of the Secondary Outcomes

**Figure 4:**
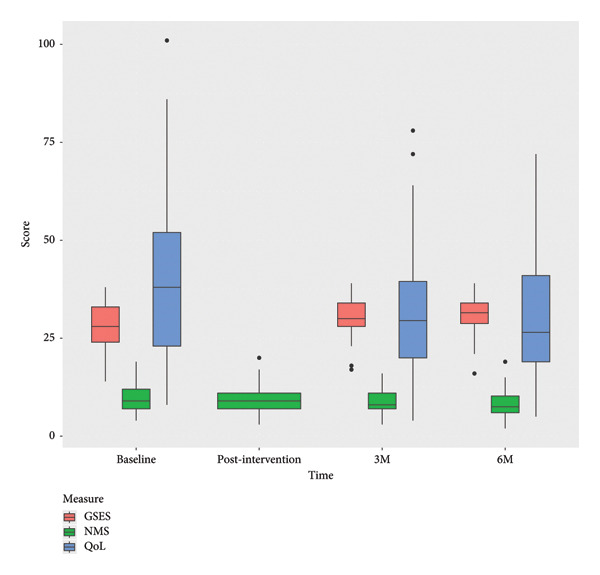
Boxplots of the clinical outcome measures, GSES, PDQ‐39, and NMS at baseline and 3‐ and 6‐month follow‐ups.

Secondary outcomes were assessed at baseline and 3‐ and 6‐month follow‐ups and showed positive trends in QoL, as the change in QoL score from baseline to the 3‐month follow‐up was significant (*p* = 0.001); however, the improvement was not statistically significant from baseline to 6‐month follow‐up (*p* = 0.072). This indicates that the effect levels off on a long‐term basis (Figure 4). No change in NMS burden was observed (*p* = 0.465). GSES improved significantly from baseline to 6‐month follow‐up (*p* = 0.026).

**Figure 5:**
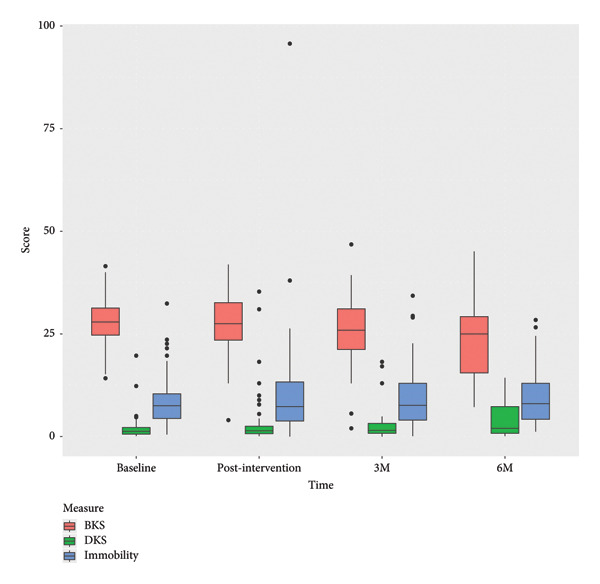
Boxplots of the PKG data on changes in motor symptoms, bradykinesia score (BKS), dyskinesia score (DKS), and time with immobility.

The analyses showed improvements in motor symptom scores from baseline to postintervention and at follow‐up, based on objective data from the PKG watch (Figure 5). The difference was statistically significant in BKS (*p* = 0.0117) and DKS which increased (*p* = 0.0015). The change in time with immobility scores was not statistically significant (*p* = 0.513).

**Figure 6:**
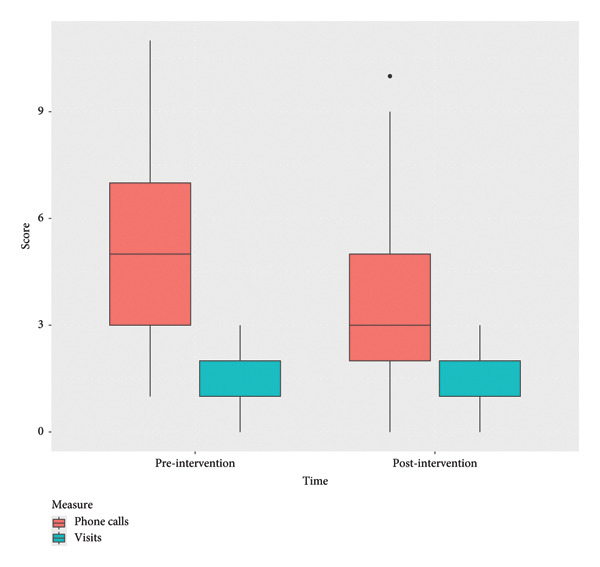
Boxplots of the change in telephone contacts and visits to the MDC 6 months preintervention and 6 months postintervention.

The analyses also included differences in number of contacts to the MD clinics. The boxplots show the distribution of the number of phone calls and visits both 6 months pre‐intervention and 6 months post‐intervention (Figure 6).

Pre‐intervention, the mean number of contacts (phone calls) was noticeably higher, with a broader distribution, indicating more variability in the number of contacts (mean = 5.4, range 2–11). Post‐intervention, the number of telephone contacts significantly decreased, showing a more compact distribution and lower mean scores (mean = 3.1, range 0–9, *p* = 0.0036).

Pre‐ and post‐intervention distributions for physical consultations at the MDCs had no substantial change in the median or variability and are consistent with the nonsignificant *p* value = 0.154.

## 4. Discussion

The results of this long‐term follow‐up study show a positive trend that the 8‐week self‐management program may be effective in empowering individuals with PD to take a more active role in managing their symptoms, with sustained improvements in health literacy, impact of the program on self‐management strategies, and QoL, along with statistically significant improvement in self‐efficacy, reductions in BKS, and the frequency of telephone contacts with the clinics.

The cohort in this study exhibited a relatively high level of education, with 72.5% of participants reporting either higher education (43.7%) or a graduate degree (28.8%). This demographic profile is important to consider when interpreting the HeiQ and HL scores, as education is a well‐established determinant of HL across various populations, including those with chronic illnesses such as PD [29]. In this cohort, the elevated HL scores may reflect this educational advantage. Our catchment areas included mixed socioeconomic neighborhoods, and no educational criteria were applied. However, despite a trend toward improvement in both parts of the HLQ‐14 and the HeiQ instrument following the intervention, neither of the scores showed statistically significant within‐subject changes. This lack of statistically significant change, despite a high educational background, may be due to a ceiling effect, meaning that the participants may have already had high baseline HL scores due to their education level and relatively high cognition level, leaving limited room for measurable improvement. Moreover, the numbers may reflect participation bias and could limit generalizability to PwP with lower health literacy. Future implementations should include purposeful recruitment and assisted data collection to better represent underserved groups.

The reduction in numbers of telephone contacts to the clinic 6 months after the intervention suggests that the program can reduce the need of support from clinical healthcare. Data from the evaluation survey collected post‐intervention (before follow‐up measurements) show that the reduced need of support was typically in terms of adherence to medication, management of symptoms, and better recognition of nonrelated PD symptoms. Overall, our results indicate improved efficiency in managing self‐care and suggest that remote monitoring and self‐management strategies can reduce the burden on healthcare systems without compromising patient health outcomes, which is in line with similar studies examining PD and self‐management [30, 31]. One of the studies suggests that self‐management focus in combination with a remote monitoring system can motivate patients to increase activity levels, potentially reducing the need for in‐person consultations within, e.g., physiotherapy [31]. Surprisingly, our results did not show a reduction in time with immobility based on PKG data, despite most participants using these scores as motivation to be more active throughout the program.

Further, the use of the PKG watch enabled objective monitoring of motor symptoms and provided the participants with insights into individual motor symptom profile. Notably, the participants themselves leveraged PKG‐generated reports during clinical consultations in the project period to optimize medical management, verify treatment appropriateness, and guide decisions on advanced therapeutic interventions in dialog with their neurologist. This suggests that access to objective measurements, like PKG data, can empower PwP to take a more active role in their care, as seeing their own scores and fluctuations helped participants better understand their condition and contribute to more informed consultations with clinicians. The average BK score decreased significantly but the DKS on the other hand increased, which raises the question of whether adapted strategies from the program were driven by improved symptom self‐management or adjustments in treatment strategies informed by PKG data. Dorsey et al. explored how wearable technologies can not only enhance the precision of symptom monitoring but also that real‐time feedback from wearable devices fosters a sense of control, enabling patients to identify symptom patterns, seek timely medical advice, and motivate PwP to engage in modifying regimens [32]. This can explain why the participants on average improved significantly in their BK score. However, as outlined, in a systematic review by Ancona et al. [33], more research examining the practicality and implementation of wearable technology and how to use this as a support tool in dialog with PwP and caregivers is needed. It may also require a shift of attitudes among clinicians and more validation on how to integrate wearable data into clinical practice [34, 35].

Additionally, sleep fragmentation and sleep quality data from the PKG were also available by means of 24 h of recording [36], and combined with a focus on sleep disturbances in the group sessions, it helped many of the participants to understand their individual sleep patterns and integrate modifying strategies. Based on the survey feedback from the participants, the objective data and the small workshop discussions clarified many PD‐related issues to spouses in terms of the night sleep and helped the parties to create a dialog about acting out dreams and activity during the night time. This, in combination with the mindfulness sessions creating more mental health, could have an impact on the positive trend in QoL during the intervention. Overall, the program element with individual goal‐setting particularly focused on sleep disturbances and other NMS, as they had the most negative effect on daily life among the participants. However, the lack of significant changes in total NMS burden among the participants aligns with the understanding that managing these symptoms is complex and requires specialized expertise due to their broad spectrum [37]. The PD GOAL instrument [17] was vital in raising participants’ awareness of both motor symptoms and NMS, encouraging proactive management. In other studies, it is highlighted that increased awareness empowers patients to seek timely help for issues like depression or sleep problems and recognize their PD‐related nature [37, 38], which is a critical first step in addressing them.

The program emphasizes the need of shared responsibility of both HCPs and patients in managing PD, with a strong emphasis on self‐management strategies in a nonpharmacological intervention [39]. Thus, the impact of the co‐developed program on empowering participants cannot be attributed to a single component. Instead, the program’s effect should be viewed as a complementary “self‐management package,” where all included elements are interconnected and contribute to a broader overall goal in addressing the complexities of PD, rather than the result of any isolated element.

### 4.1. Strengths, Limitations, and Future Directions

A co‐creation approach was used to develop this self‐management program, which may foster a greater sense of meaningfulness and relevance of the program, leveraging the diverse perspectives of the “end‐users,” the PwP, and caregivers in a collaborative approach. Because caregiver attendance varied and caregiver‐reported outcomes were not collected, unmeasured effects of caregiver participation may persist and could confound observed patient outcomes; however, the role of the caregiver cannot be separated from daily self‐management strategies.

A large body of the results did not reach statistical significance, even though positive trends were observed. Future research involving larger cohorts and longer follow‐up periods is essential to validate the findings and evaluate the program’s long‐term impact. Additionally, integrating objective tools like the PKG watch into routine clinical practice shows promise for enhancing personalized care contributing to a more equal and dynamic interaction with PwP in managing the disease and finding the right treatment. However, further studies are needed to assess its implementation feasibility and clinical effectiveness in terms of an empowerment tool.

Generalizability is limited by the exclusion of H&Y stages 4–5. Although a pilot inclusion of stage‐4 participants was attempted, recruitment barriers prevented adequate enrollment. Future iterations should consider home‐based/telehealth delivery, caregiver‐mediated formats, and flexible scheduling to feasibly include later‐stage PD. Additionally, an online self‐management program has been developed and implemented in MDCs nationally in Denmark [40], from which PwP in late‐stage PD can benefit via caregiver‐mediated participation and tailored content.

## 5. Conclusion

This long‐term follow‐up study suggests that the 8‐week self‐management program can empower individuals with PD to take a more active role in managing their symptoms, fostering trends of improvements in health literacy, QoL, and symptom management, sustained over 6 months, with statistically significant improvements in reduction of motor symptoms and telephone contacts to clinics and improvement in self‐efficacy. The reduction in telephone contacts with the MDCs highlights the program’s potential to enhance healthcare efficiency by leveraging patients with tools of day‐to‐day management. This study also highlights the potential usefulness of integrating home‐based wearable data into an empowerment approach to provide patients with actionable insights, enhance symptom tracking, and further support personalized self‐management strategies. While the findings are promising, further research with larger cohorts and longer follow‐up is necessary to confirm the program’s long‐term benefits and broader clinical applicability.

## Ethics Statement

The study was granted ethical approval from the Capitol Region Research Ethics Committee, Copenhagen (IRB: H‐22013230) and was registered on the data registration portal, *Privacy* (P‐2021‐796).

## Conflicts of Interest

The authors declare no conflicts of interest.

## Funding

Trine Hørmann Thomsen has received funding from Jascha Foundation. The Novo Nordisk Foundation and Capitol Region Research Foundation provided funding to this study.

## Supporting Information

Figure S1: the co‐developed PD GOAL instrument used by the participants in their individual goal‐setting.

## Supporting information


**Supporting Information** Additional supporting information can be found online in the Supporting Information section.

## Data Availability

The datasets generated and analyzed during the current study are available from the corresponding author upon reasonable request.
