# Supplementary material for: EMPOWER‐PD: Evaluation of an 8‐Week Co‐Designed Self‐Management Program in People With Parkinson’s Disease and Their Caregivers: An Intervention Study With Long‐Term Follow‐Up
Source: Parkinsons Dis. 2025 Dec 6;2025:9413261. doi: 10.1155/padi/9413261 (PMC12767402; doi:10.1155/padi/9413261)
Supplement: Supplementary file 1 — Supporting Information Additional supporting information can be found online in the Supporting Information section. [file PADI-2025-9413261-s001.pdf]

Coping in Everyday Life

” PD-GOAL”

Write down the 3 most bothersome symptoms: NRS (0-10, 10=most bothersome)

1.. | 0 | 1 | 2 | 3 | 4 | 5 | 6 | 7 | 8 | 9 | 10 |

2.. | 0 | 1 | 2 | 3 | 4 | 5 | 6 | 7 | 8 | 9 | 10 |

3.. | 0 | 1 | 2 | 3 | 4 | 5 | 6 | 7 | 8 | 9 | 10 |

Identify current coping strategies from everyday life that helps you manage your symptoms.

____

Identify a personal goal(s) that you want to or hope to be able to manage based on the most bothersome symptom/challenge, and clarify potential strategies and motivational factors:

Symptom/challenge	GOAL	Coping strategy	Motivational factors
Example: Isolation in social activities	*To manage the visibility of my symptoms in a better way*	*Be more open to other people about my disease-related changes*	*Support from my family and friends (feedback and/or emotional support)*

When do you have the most energy during the day (less symptoms)?

00	01	02	03	04	05	06	07	08	09	10	11	12	13	14	15	16	17	18	19	20	21	22	23

Evaluation:

Which goal(s) do you want to work with in the future?:

____

New goals?

____

Figure S1: *The PD-GOAL instrument was initially developed through a co-creation process and was actively used by the participants in the self-management program during the intervention*.
